# Supplementary material for: A regeneration-triggered metabolic adaptation is necessary for cell identity transitions and cell cycle re-entry to support blastema formation and bone regeneration
Source: eLife. 2022 Aug 22;11:e76987. doi: 10.7554/eLife.76987 (PMC9395193; doi:10.7554/eLife.76987)
Supplement: Supplementary file 2. [file elife-76987-supp2.docx]

**Supplementary File 2a:** Primer sequences for q-PCR assay.

| Gene | Forward Primer  (5’>3’) | TM (ºC) | Reverse Primer  (5’>3’) | TM (ºC) |
| --- | --- | --- | --- | --- |
| *ef1a*  ENSDARG00000039502 | CCTGGGAGTGAAACAGCTG | 63.6 | GCCTCCAGCATGTTGTCAC | 64.6 |
| *hk1*  ENSDARG00000039452 | CCGTGGACTGGATCATCTG | 60.1 | TTACCGCTTCCGTCTTCTGA | 60.9 |
| *hk2*  ENSDARG00000101482 | GCCTCAATCAGCTCAGCAT | 62.1 | CTGACTGGACGAGACTGACCT | 62.5 |
| *Pfkpa*  ENSDARG00000028000 | CAGAAGACTCGGCCTGTTTG | 64.9 | GCAACTTCAGCCACCACTG | 64.7 |
| *Aldoaa*  ENSDARG00000011665 | CTCAATGCCATGAACCAGTG | 64.2 | GGCCTGGCTGTTGTTAAGAG | 63.7 |
| *Gapdhs*  ENSDARG00000039914 | CCAATGAAGGGAATTCTGGG | 65.3 | CAGGTCAGCAACACGATGG | 65.6 |
| *pgam1A*  ENSDARG00000005423 | TGAGAGGCATTGTGAAGCAC | 64.1 | CTTTGCGAACGGTTTCCTC | 64.6 |
| *pgam1B*  ENSDARG00000014068 | GCAGATCAAGGAGGGAAAGAG | 64.1 | GGCTTCAGGTTCTTGTCCAG | 63.8 |
| *eno1a*  ENSDARG00000022456 | TCACCGTTCTGGAGAGACG | 64.3 | GAAGCGAGCCTTGTCTCCT | 63.7 |
| *pkma*  ENSDARG00000099730 | CGACAGGCTCATCTTTACCG | 63.3 | CCACATCACCAGCCTTGAA | 63.1 |
| *Ldha*  ENSDARG00000101251 | GTCAAGGGAATGCATGGTG | 64.1 | CTGAACACCCCACAAGGTC | 63.2 |
| *pdha1a*  ENSDARG00000012387 | CACGTGAAGGAGATCCAGGA | 61.2 | CCTTACCTCCACATCAATCTCC | 62.8 |
| *pdha1b*  ENSDARG00000010555 | TGATTAGCAGCAACATGGCCA | 64.8 | GCCTCTGACCTCTAATGGAGCA | 63.0 |
| *dlat*  ENSDARG00000015918 | GGCATGTATGGCATCAAGC | 64.1 | GATCGCAGCTCAGAGTCACA | 64.6 |
| *Sdhdb*  ENSDARG00000030139 | TCTTCTGAGCCTGGCACC | 64.4 | GACAGAACAAACAGGCCTGC | 64.8 |
| *mdh2*  ENSDARG00000043371 | GAGCCAGGTTCACATTCTCC | 63.7 | CCCAAGGCCAAGGTTCTTT | 65.1 |
| *ndufv2*  ENSDARG00000013044 | CGATGGTCCAAATCAACG | 62.6 | CAGGTCAGCTCTCACACCA | 62.9 |
| *cox6c*  ENSDARG00000038577 | TGCGTTTGCTCTTTCCCTC | 65.4 | GGCCTGGCACTTTCAAAGAT | 65.3 |
| *coa3*  ENSDARG00000100585 | ATGGCGCAGTGGAAGAAG | 64.1 | CGGTACACGCACTTTTGC | 63 |
| *coa5*  ENSDARG00000094833 | AGCCTGTCTCCTTCAGCATGA | 66.3 | TGCCTCTGAATCGAGATCG | 65 |
| *runx2a*  ENSDARG00000040261 | ACGGTAATGGCTGGAAATGA | 64.1 | GTCCGTCCACTGTGACCTTT | 64.1 |
| *runx2b*  ENSDARG00000059233 | AGCTTCACCCTGACGATTACA | 63.5 | CCAGTTCACTGAGACGGTCA | 64.1 |
| *osx*  ENSDARG00 000019516 | TCCAGACCTCCAGTGTTTCC | 64.2 | ATGGACATCCCACCAAGAAG | 63.8 |
| *col10a1*  ENSDARG0000 0054753 | GCATTCTTCTTCTCCTGGTG | 61.4 | CCTGAACCCCAACCCCC | 67.7 |
| *bglp*  ENSDARG00000058414 | TGACGTGGCCTCTATCATCA | 64.4 | TTTATAGGCGGCGATGATTC | 63.6 |
| *bglpl*  ENSDARG00000104467 | AACTCTGCCAGTGCTGAAGG | 64.6 | GGTCTCAGCCATGTGTTCAC | 63.3 |
| *osn*  ENSDARG00000019353 | GGTCGTGGAGGATGTTATTGC | 65.4 | GGGGCAGGTCAAAGGGTC | 66.7 |
| *cyp26b1*  ENSDARG00000077121 | CTCCAATCCTGACCCCATCAA | 68.2 | GCAGGTCGATGGGAAGACTGA | 68.8 |
| *bmp2a*  ENSDARG00000013409 | ATCAGGAGCTTCCACCATGA | 64.7 | TGAACGTTAATGCGGTGAAA | 63.9 |
| *bmp2b*  ENSDARG00000041430 | CTGAAAACGATGACCCGAAC | 64.4 | AACTGCTGCGTTGTTTTTCC | 64 |
| *nfkbiaa*  ENSDARG00000005481 | CCGTAGACCTGCAGAACCTG | 60.8 | CTCGCTGTTGTGTCTGCC | 62.1 |
| *Nfkbiab*  ENSDARG00000007693 | GTCGCCATCCAGGGTTACTT | 61.3 | ACACTGTGAACACTGGCACC | 62.6 |
| *drp1*  ENSDARG00000015006 | TTCCTGGTGAACCACGTGAAG | 63.4 | CCTGACTGGCTTTCTGCAAGG | 64.5 |
| *fis1*  ENSDART00000135509 | GAACCGGACAACAAACAGG | 63.00 | CGGCCAAACCAATAAGACC | 64.10 |
| *mtfr1*  ENSDART00000066628 | TGAACCCACAGATGCAGC | 63.8 | CAAACAGCGGTGTTTCCAC | 64.2 |

| Antibody | Host | Dilution | Localization | Company |
| --- | --- | --- | --- | --- |
| Anti-PCNA | Rabbit | 1:100 | Nucleus; G1-proliferating Cells | Sta Cruz Biotechnology,  F2007 |
| Anti-Runx2 | Mouse | 1:50 | Nucleus; basal expression in mature OB and high expression in pre-Obs and immature OB | Sta Cruz Biotechnology,  101145 |
| Anti-ZNS5 | Mouse | 1:200 | Membrane; expressed in all stages of OB lineage inclusive in joint-associated OP | Zebrafish International Resource Centre, 011604 |
| Anti-  Ds-Red/  mCherry | Rabbit | 1:200 | mCherry expressing cells | Clontech,Takara 632496 |
| Anti-GFP | Mouse | 1:100 | GFP expressing cells | Roche, 11814460001 |
| Anti-GFP | Rabbit | 1:100 | GFP expressing cells | Life technologies, A11122 |

**Supplementary File 2b:** List of primary antibodies used for immunofluorescence assays.

| Fluorophore | Host | Specificity | Dilution | Company |
| --- | --- | --- | --- | --- |
| Alexa Fluor 488 | Goat | Mouse | 1:500 | Invitrogen,  A11001 |
| Alexa Fluor 488 | Goat | Rabbit | 1:500 | Invitrogen,  A11070 |
| Alexa Fluor 568 | Goat | Mouse | 1:500 | Invitrogen,  A11031 |
| Alexa Fluor 568 | Goat | Rabbit | 1:500 | Invitrogen,  A11036 |
| Cy5 | Goat | Mouse | 1:250 | Invitrogen,  A10524 |
| Alexa Fluor 647 | Donkey | rabbit | 1:500 | Jakson Imuno Research  711605152 |

**Supplementary File 2c:** List of primary antibodies used for immunofluorescence assays.

**Supplementary File 2d:** List of sample size number and statistical test preformed for each quantitative experimental design.

| Figure | Sample size (n) | | Statistical test |
| --- | --- | --- | --- |
| Main Figures | | | |
| Fig 1B | | 4 cDNA pools per time-point | Paired t-test |
| Fig 1D | | 0hpa= 18, 5hpa= 8, 10hpa= 16, 15hpa= 9, 20hpa= 9,25hpa= 9 bony-rays from 3 different fish specimens per condition | Kruskal-Wallis for multiple comparisons |
| Fig 1E | | 0hpa= 6, 6hpa= 10, 12hpa= 12, 18hpa= 9, 24hpa= 13 cryosections from 3 different fish specimens per time-point | Kruskal-Wallis for multiple comparisons |
| Fig 1H | | 0hpa= 27, 12hpa= 26, 24hpa= 20 cryosections from 4-5 blastemas from 4 different fish specimens per time-point | Kruskal-Wallis for multiple comparisons |
| Fig 1I | | 0hpa= 27, 12hpa= 26, 24hpa= 18 cryosections representing 4-5 blastemas from 4 different fish specimens per time-point | Kruskal-Wallis for multiple comparisons |
| Fig 2B | | 3 cDNA pools per time-point | t-test with Welch’s correction |
| Fig 2C | | 5 cDNA pools per time-point | Paired t-test |
| Fig 2D | | 4 cDNA pools per time-point | Paired t-test |
| Fig 2E | | 4 metabolite pools per time-point | Mann-Whitney |
| Fig 2N | | 0hpa=59, 24hpa=83 bony-rays from 3 and 4 different fish specimens, respectively | Mann-Whitney |
| Fig 3C | | 0 hpa: PBS=7, 2DG=8; 0-12hpa: PBS=6, 2DG=7; 0-24hpa: PBS=4, 2DG=5; 0-36hpa: PBS=5, 2DG=6 fish per condition | Mann-Whitney |
| Fig 3O | | 0-36hpa: PBS=13, SO=12 fish per condition | Mann-Whitney |
| Fig 3S | | 0-36hpa: PBS:DMSO(1:1)/UK5099=11 fish per condition | Mann-Whitney |
| Fig 4C | | 8 cDNA pools per condition | Paired t-test |
| Fig 4F | | 0-12hpa: PBS=79, 2DG=89 cryosections representing 5-6 blastemas from 6 different fish specimens per condition | Mann-Whitney |
| Fig 4G | | 0-12hpa: PBS=79, 2DG=89 cryosections representing 5-6 blastemas from 6 different fish specimens per condition | Mann-Whitney |
| Fig 5E | | 0-12hpa: PBS=90, 2DG=82 bony-rays from 4 different fish specimens | Mann-Whitney |
| Fig 5H | | 0-12hpa: PBS=23, 2DG=30 cryosections representing 4-6 blastemas from 4 and 6 different fish specimens per condition, respectively | Mann-Whitney |
| Fig 5I | | 0-12hpa: PBS=23, 2DG=27 cryosections representing 4-6 blastemas from 4 and 6 different fish specimens per condition, respectively | Mann-Whitney |
| Fig 5J | | 10 cDNA pools per condition | Paired t-test |
| Fig 6E | | 0-36hpa: PBS=27, 2DG=25 cryosections representing 4-6 blastemas from 6 and 5 different fish specimens per condition, respectively | Mann-Whitney |
| Fig 6F | | 0-36hpa: PBS=27, 2DG=25 cryosections representing 4-6 blastemas from 6 and 5 different fish specimens per condition, respectively | Mann-Whitney |
| Fig 6G | | 0-36hpa: PBS=27, 2DG=25 cryosections representing 4-6 blastemas from 6 and 5 different fish specimens per condition, respectively | Mann-Whitney |
| Fig 6J | | 0-36hpa: PBS=28, 2DG=27 cryosections representing 4-5 blastemas from 6 and 5 different fish specimens per condition, respectively | Mann-Whitney |
| Fig 6K | | 0-36hpa: PBS=27, 2DG=27 cryosections representing 4-5 blastemas from 6 and 5 different fish specimens per condition, respectively | Mann-Whitney |
| Supplement Figures | | | |
| Figure 2 - figure supplement 2A | | 6 cDNA pools per time-point | Paired t-test |
| Figure 2 -figure supplement 2B | | 4 cDNA pools per time-point | Paired t-test |
| Figure 2 -figure supplement 2I | | 0hpa/ 6hpa/ 24 hpa= 13 cryosections representing 3 blastemas from 5 different fish specimens per time-point | Mann-Whitney |
| Figure 2 -figure supplement 2J | | 0hpa/ 6hpa/ 24 hpa= 12 cryosections representing 3 blastemas from 3 different fish specimens per time-point | Mann-Whitney |
| Figure 3 -figure supplement 1D | | 0-36hpa: DMSO=15, 3PO=14 fish per condition | Mann-Whitney |
| Figure 3 -figure supplement 1H | | 0-36hpa: DMSO=4, MB6=4 fish per condition | Mann-Whitney |
| Figure 5 -figure supplement 1G | | 0-12hpa: PBS=26, 2DG=35 cryosections representing 4-6 blastemas from 5 and 6 different fish specimens per condition, respectively | Mann-Whitney |
| Figure 5 -figure supplement 1H | | 0-12hpa: PBS=26, 2DG=35 cryosections representing 4-6 blastemas from 5 and 6 different fish specimens per condition, respectively | Mann-Whitney |
| Figure 5 -figure supplement 1O | | 24hpa: DMSO=10, 3PO=14 cryosections representing 3-4 blastemas from 3 and 4 different fish specimens per condition, respectively | Mann-Whitney |
| Figure 5 -figure supplement 2I | | 0-12hpa: PBS=17, 2DG=16 cryosections representing 4-6 blastemas from 4 and 3 different fish specimens per condition, respectively | Mann-Whitney |
| Figure 5 -figure supplement 2J | | 0-12hpa: PBS=17, 2DG=16 cryosections representing 4-6 blastemas from 4 and 3 different fish specimens per condition, respectively | Mann-Whitney |
| Figure 5 -figure supplement 2K | | 0-12hpa: PBS=21, 2DG=18 cryosections representing 4-6 blastemas from 4 and 3 different fish specimens per condition, respectively | Mann-Whitney |
| Figure 5 -figure supplement 2L | | 0-12hpa: PBS=21, 2DG=18 cryosections representing 4-6 blastemas from 4 and 3 different fish specimens per condition, respectively | Mann-Whitney |
| Figure 6 -figure supplement 1K | | 24hpa: DMSO=18, 3PO=19 cryosections representing 3-5 blastemas from 4 and 5 different fish specimens per condition, respectively | Mann-Whitney |
| Figure 6 -figure supplement 1L | | 24hpa: DMSO=18, 3PO=19 cryosections representing 3-5 blastemas from 4 and 5 different fish specimens per condition, respectively | Mann-Whitney |
| Figure 6 -figure supplement 2G | | 0-12hpa: PBS=25, 2DG=29 cryosections representing 4-6 blastemas from 5 different fish specimens per condition | Mann-Whitney |
| Figure 6 -figure supplement 2H | | 0-12hpa: PBS=25, 2DG=29 cryosections representing 4-6 blastemas from 5 different fish specimens per condition | Mann-Whitney |
| Figure 6 -figure supplement 3C | | 0-36hpa: PBS=26, 2DG=32 cryosections representing 4 blastemas from 5-6 different fish specimens per condition | Mann-Whitney |
| Figure 6 -figure supplement 4L | | 0-6dpa: PBS=5, 2DG=6 fish specimens | Mann-Whitney |
| Figure 6 -figure supplement 4N | | 0-6dpt: PBS=5, 2DG=6 fish representing 15-18 bony rays per fish | Mann-Whitney |
