# Supplementary material for: A regeneration-triggered metabolic adaptation is necessary for cell identity transitions and cell cycle re-entry to support blastema formation and bone regeneration
Source: eLife. 2022 Aug 22;11:e76987. doi: 10.7554/eLife.76987 (PMC9395193; doi:10.7554/eLife.76987)
Supplement: Transparent reporting form [file elife-76987-transrepform1.docx]

***eLife’s* transparent reporting form**

We encourage authors to provide detailed information *within their submission* to facilitate the interpretation and replication of experiments. Authors can upload supporting documentation to indicate the use of appropriate reporting guidelines for health-related research (see [EQUATOR Network](http://www.equator-network.org/%20)), life science research (see the [BioSharing Information Resource](https://biosharing.org/)), or the [ARRIVE guidelines](http://www.plosbiology.org/article/info:doi/10.1371/journal.pbio.1000412) for reporting work involving animal research. Where applicable, authors should refer to any relevant reporting standards documents in this form.

If you have any questions, please consult our Journal Policies and/or contact us: [editorial@elifesciences.org](mailto:editorial@elifesciences.org).

**Sample-size estimation**

- You should state whether an appropriate sample size was computed when the study was being designed
- You should state the statistical method of sample size computation and any required assumptions
- If no explicit power analysis was used, you should describe how you decided what sample (replicate) size (number) to use

Please outline where this information can be found within the submission (e.g., sections or figure legends), or explain why this information doesn’t apply to your submission:

No computation analysis was used to determine sample size. Experimental designs aimed to have at least 4-5 animals per condition. For immunofluorescent analysis 3-5 animals were used per condition and, cryosections representing 3-4 blastemas per animal were analysed. In this case blastemas are used as independent unites, which is in accordance with the literature.

Information regarding sample size used for each experiment can be found in the Material and Methods section, in figure legends and, the detailed description in Supp table 4.

**Replicates**

- You should report how often each experiment was performed
- You should include a definition of biological versus technical replication
- The data obtained should be provided and sufficient information should be provided to indicate the number of independent biological and/or technical replicates
- If you encountered any outliers, you should describe how these were handled
- Criteria for exclusion/inclusion of data should be clearly stated
- High-throughput sequence data should be uploaded before submission, with a private link for reviewers provided (these are available from both GEO and ArrayExpress)

Please outline where this information can be found within the submission (e.g., sections or figure legends), or explain why this information doesn’t apply to your submission:

This information can be found in the material and methods section for the gene expression experiments (qPCR and Osteoblast ArrayXS).

For the Osteoblast ArrayXS a GEO access number was provided in the submission form.

**Statistical reporting**

- Statistical analysis methods should be described and justified
- Raw data should be presented in figures whenever informative to do so (typically when N per group is less than 10)
- For each experiment, you should identify the statistical tests used, exact values of N, definitions of center, methods of multiple test correction, and dispersion and precision measures (e.g., mean, median, SD, SEM, confidence intervals; and, for the major substantive results, a measure of effect size (e.g., Pearson's r, Cohen's d)
- Report exact p-values wherever possible alongside the summary statistics and 95% confidence intervals. These should be reported for all key questions and not only when the p-value is less than 0.05.

Please outline where this information can be found within the submission (e.g., sections or figure legends), or explain why this information doesn’t apply to your submission:

For each experiment statistical tests, N values, dispersion and precision measures are display on graphs in figure and supplementary figure legends. A resume of this information is also provided in Supp table 5.

(For large datasets, or papers with a very large number of statistical tests, you may upload a single table file with tests, Ns, etc., with reference to sections in the manuscript.)

**Group allocation**

- Indicate how samples were allocated into experimental groups (in the case of clinical studies, please specify allocation to treatment method); if randomization was used, please also state if was applied
- Indicate if masking was used during group allocation, data collection and/or data analysis

Please outline where this information can be found within the submission (e.g., sections or figure legends), or explain why this information doesn’t apply to your submission:

For sample allocation into experimental groups, we tried to achieve balance between study groups by using same size, same proportion between sexes and animals with similar weights and ages. This was particularly relevant for qPCR experiments as control and treated animals are analysed using paired tests.

Due to the severe caudal fin morphological differences between controls and treated animals the experimental procedures could not be properly masked.

**Additional data files (“source data”)**

- We encourage you to upload relevant additional data files, such as numerical data that are represented as a graph in a figure, or as a summary table
- Where provided, these should be in the most useful format, and they can be uploaded as “Source data” files linked to a main figure or table
- Include model definition files including the full list of parameters used
- Include code used for data analysis (e.g., R, MatLab)
- Avoid stating that data files are “available upon request”

Please indicate the figures or tables for which source data files have been provided:

Microarray data have been deposited in GEO under accession code GSE194385

Other raw data files and MATLAB scripts for the following figures was uploaded to the eLife website:

Fig1; Fig2; Fig3; Fig4, Fig5; Fig6; Supp Fig2; Supp Fig3; Supp Fig4; Supp Fig5; Supp Fig6; Supp Fig7; Supp Fig8; Supp Fig9.
